# Supplementary material for: Stabilization of β-Carotene Liposomes with Chitosan–Lactoferrin Coating System: Vesicle Properties and Anti-Inflammatory In Vitro Studies
Source: Foods. 2025 Mar 12;14(6):968. doi: 10.3390/foods14060968 (PMC11941038; doi:10.3390/foods14060968)
Supplement: Supplementary file 1 [file foods-14-00968-s001.zip › foods-3492219-supplementary.pdf]

Supplementary tables

Table S1 Composition of major phospholipid species in TH-PL.

phospholipid species	content
lysophosphatidylcholine	0.23%
lysophosphatidylethanolamine	0.03%
phosphatidylcholine	69.13%
phosphatidylethanolamine	6.61%
phosphatidylserine	0.84%
phosphoinositide	0.30%
sphingomyelin	7.93%
total	85.07%

Table S2 Preparation of Chitosan-Lactoferrin Co-Modified Liposome System with Various Substance Ratios

Types of substances	Concentration (mg/mL)
Fish head phospholipids	1.0
Cholesterol	0.2
β-carotene loading capacity	0.01
Chitosan	0.8
Lactoferrin	0.375
